# Guilt, tears and burnout—Impact of UK care home restrictions on the mental well‐being of staff, families and residents

**DOI:** 10.1111/jan.15181

**Published:** 2022-02-21

**Authors:** Clarissa Giebel, Kerry Hanna, Paul Marlow, Jacqueline Cannon, Hilary Tetlow, Justine Shenton, Thomas Faulkner, Manoj Rajagopal, Stephen Mason, Mark Gabbay

**Affiliations:** ^1^ Department of Primary Care & Mental Health University of Liverpool Liverpool UK; ^2^ NIHR ARC NWC Liverpool UK; ^3^ School of Health Sciences University of Liverpool Liverpool UK; ^4^ Lewy Body Society Wigan UK; ^5^ SURF Liverpool Liverpool UK; ^6^ Sefton Older People's Forum, Sefton Liverpool UK; ^7^ Mersey Care NHS Trust Liverpool UK; ^8^ Lancashire & South Cumbria NHS Trust Lancaster UK; ^9^ Institute of Life Course and Medical Sciences University of Liverpool Liverpool UK

**Keywords:** care homes, carers, COVID‐19, long‐term care, mental health, nursing, nursing homes, staff well‐being

## Abstract

**Aims:**

The aim of this study was to explore the impact of the pandemic on the emotional and mental well‐being of family carers, care home staff and residents, in light of changing restrictions, increased testing and vaccination rollout in the UK.

**Design:**

Longitudinal, qualitative semi‐structured interview study.

**Methods:**

Remote semi‐structured interviews were conducted with family carers of care home residents with dementia and care home staff from different care homes across the UK. Baseline and follow‐up interviews were conducted in October/November 2020 and March 2021, respectively. Data were analysed using inductive thematic analysis involving members of the public with caring experiences.

**Results:**

In all, 42 family carers and care home staff participated at baseline, with 20 family carers and staff followed up. We identified four themes: (1) Developing anger and frustration; (2) Impact on relationships; (3) Stress and burnout; and (4) Behavioural changes, and perceived impact on residents. The mental health of everyone involved, including family carers, care home staff and residents, has been negatively affected, and relationships between family carers and staff have been severely strained. There was a general lack of adequate mental health support, with little relief.

**Conclusions:**

The pandemic has had a detrimental impact on the lives of those surrounding care homes—from residents and staff to family carers. Consideration should be given on how to best support the mental health needs of all three groups, by providing adequate easily accessible mental health care for all. This should also focus on rebuilding the relationships between family carers and care home staff.

**Impact:**

This is the first paper to highlight the effects of the long‐lasting and miscommunicated restrictions on residents, carers and care home staff, and highlight the urgent need for continued mental health support.

## 1 INTRODUCTION

Care home residents have been particularly adversely affected by the COVID‐19 pandemic. During the peak of the first UK pandemic wave, around a third of COVID‐19‐related deaths were care home residents (ONS, 2020). Nearly half a million people live in care homes across the UK (Department of Health, 2020; ONS, 2020; Public Health Scotland, 2020), approximately 70% of whom are living with dementia.

### 1.1 Background

Poor and under‐supported mental health and well‐being in family carers and staff has been of concern prior to the pandemic. Many family carers experience increased carer burden, particularly in the more advanced stages when symptoms and support needs increase (Sutcliffe et al., 2017; Watson et al., 2019). When relatives move into a care home, this can often be accompanied by heightened levels of depressive symptoms and feelings of guilt in family carers (Sury et al., 2013). Similarly, care home staff in the UK is working under difficult and unsupported conditions, facing difficult job demands regardless of the pandemic situation. Recent research has highlighted that burnout in care home staff varies by different demographic factors, with younger staff generally experiencing higher burnout (Costello et al., 2020), while a systematic review and meta‐analysis evidenced low to medium stress and burnout across care home staff in general (Costello et al., 2019).

While research into the effects of the pandemic on people living with dementia and unpaid carers is emerging, ranging from faster dementia deterioration, social support service shortages and increased carer burden (Cohen et al., 2020; Talbot & Briggs, 2021; Tujit et al., 2021), there has been little focus on the impact on family members of care home residents to date. O'Caoimh et al. (2020) surveyed family members of care home residents in Ireland, reporting reduced emotional well‐being linked to visiting restrictions, as well as poor levels of communication from care homes towards family members. Research from Finland further supports these findings, highlighting the emotional burden experienced by family carers and the increased sudden progression of dementia symptoms in care home residents (Paananen et al., 2021). This is corroborated by research into the mental health and emotional well‐being of unpaid carers and specifically those caring for community‐residing people living with dementia (Giebel et al., 2021; Rainero et al., 2021; Savla et al., 2020). Findings from the UCL COVID‐19 Social Study indicate higher levels of depression and anxiety in unpaid carers in the UK at all time points throughout the pandemic (Mak et al., 2021). In support of this, Hanna et al. (2021) reported increased levels of anxiety and depression, as well as loneliness in unpaid dementia carers during lockdown. However, the limited published evidence on family carer mental well‐being of care home residents predates vaccination programmes, and is country specific at present. It is important to understand the mental health impacts of the pandemic on family members, as well as residents, throughout the different stages of the pandemic in order that future care can be better orientated to serve both the residents, and their families.

The effects of the pandemic on staff working in care homes have received less academic attention than the impacts of the pandemic on frontline healthcare staff. While research details the mental health impact that working on the frontline of healthcare services (Gilleen et al., 2021; Wanigasooriya et al., 2020), the more limited evidence on the mental health of care home staff also highlights burnout among nursing home staff (Prados et al., 2021). In a Spanish survey of 340 care home staff, Prados et al. (2021) showed how increased hours during the pandemic also contributed to higher levels of burnout, but mediated by social support where available. However, this appears to be the only published studies to date into the mental health impact of COVID‐19 on care home staff, with no combined account of all three groups involved in the care home setting—residents, staff and family carers.

## 2 THE STUDY

### 2.1 Aims

Considering the high levels of emotional needs and support for both family carers and care home staff prior to the pandemic, and an indication of reduced mental well‐being in the general population during the pandemic, the aim of this longitudinal, qualitative study was to explore the impact of the pandemic on the emotional and mental well‐being of family carers, care home staff and residents. This was in light of the changing nature of care home restrictions, vaccinations and testing. Given the dearth of evidence into the mental health impacts of the pandemic's restrictions on family members, care home staff and residents together, this study provides significant initial findings highlighting the need for psychological support of frontline social care staff as well as those residing in, and being involved with, care homes.

### 2.2 Design

This is a longitudinal qualitative semi‐structured interview study.

### 2.3 Participants

Care home staff, staff working with care homes as part of their clinical roles and family carers (relatives) of a care home resident/person living with dementia (PLWD), aged ≥18, were eligible to take part. There were no exclusion criteria for care home staff such as length of working in the sector or other. We used convenience sampling for recruitment. The study was advertised through third sector organizations, an existing network of dementia and ageing groups, and social media. Interested participants contacted the principal investigator via email and an approved participant information sheet was emailed to them.

### 2.4 Ethical considerations

Verbal informed consent was taken before the audio‐recorded interview commenced. Ethical approval was obtained from the University of Liverpool Ethics Committee (Ref: 7626) prior to study commencement.

### 2.5 Data collection

We collected basic demographic characteristics of participants including age, gender, ethnicity, as well as relationship to the relative with dementia, dementia subtype (from family members); and, years of working in the care home sector, staff role and size of the care homes from care home staff. The interview topic guides were co‐produced with clinicians, unpaid carers of people living with dementia, and academics, and guided by the experiences of carers and professionals. Iterations of the topic guide were circulated between team members until a final version was reached. Family members were asked about their experiences of care home visitation during the pandemic, and potential impacts on their relationships with residents. Care home staff was asked about their experiences of providing care and working in care homes during the pandemic, how much support they have received with their changing work demands, and difficulties they have faced. Topic guides for baseline and follow‐up interviews are attached in Appendices I and II.

Baseline interviews were conducted between October and November 2020, and follow‐up interviews were conducted in March 2021, when COVID vaccinations were ongoing and restrictions were lifted to first allow one visitor, then two, into the care home using PPE. Two researchers with in‐depth experience of conducting qualitative researchers (CG, senior research fellow, KH, lecturer, both with PhDs) collected the data. Interviews were conducted remotely over the phone or online (Zoom), and verbal informed consent was recorded on an audio recorder prior to the interview. Interviews lasted up to 60 min.

### 2.6 Data analysis

Audio files were transcribed (and anonymized in the process), before blind double‐coding by the research team (CG, KH, SM, JC and MG) and an assistant psychologist. Recruitment ceased once data saturation was noted. Data were analysed descriptively, employing inductive thematic analysis (Braun & Clarke, 2006), and the final, conceptualized themes were discussed with carers to ensure mutual agreement. Specifically, coders met at several times after coding, and once between finalizing codes, to discuss emerging themes.

### 2.7 Validity and reliability

Data were collected by two researchers with in‐depth expertise and training in qualitative research, as outlined above. In addition, one public adviser was trained in analysing qualitative data and supported the data analysis further, enabling a broad professional and lived expertise in dementia care to analyse and interpret the findings.

### 2.8 Public involvement

One current and two former unpaid carers were involved in all stages of the study, including study document design, contribution to group discussion, and interpretation and dissemination of findings. Public involvement fees were paid according to NIHR INVOLVE (2013) guidelines.

## 3 FINDINGS

In all, 42 participants (26 family carers and 16 care home staff) were included in the baseline interviews, and 20 (*n* = 11 family carers and *n* = 9 Care home staff) took part in a follow‐up interview. Table 1 shows the demographics of the included participants. Using thematic analysis, we identified four main themes: (1) Developing anger and frustration; (2) Impact on relationships; (3) Stress and burnout; and (4) Behavioural changes, and perceived impact on residents (see Table 2). Each theme comprised of a number of sub‐themes, and both themes and subthemes are illustrated by quotes that represent typical responses for the respective themes and sub‐themes.

**TABLE 1:** Demographic characteristics of family carers and care home staff

	Family carers baseline (*n* = 26)	Family carers follow up (*n* = 11)	Care home staff baseline (*n* = 16)	Care home staff follow up (*n* = 9)	Total sample (*n* = 42)
	*N* (%)	*N* (%)	*N* (%)	*N* (%)	*N* (%)
Gender					
Female	18 (69.2%)	8 (72.7%)	13 (81.3%)	8 (88.9%)	31 (73.8%)
Male	8 (30.8%)	3 (27.3%)	3 (18.8%)	1 (11.1%)	11 (26.3%)
Ethnicity					
White British	22 (84.6%)	10 (90.9%)	13 (81.3%)	7 (77.8%)	35 (56.5%)
White Other	2 (7.7%)	1 (9.1%)	1 (6.3%)	1 (11.1%)	3 (4.8%)
BAME	2 (7.7%)	0	1 (6.3%)	0	3 (4.8%)
Prefer not to say	0	0	1 (6.3%)	1 (11.1%)	1 (1.6%)
Relationship with PLWD					
Spouse	9 (34.6%)	3 (27.3%)			
Partner	1 (3.8%)	0			
Adult child	16 (61.5)	8 (72.7%)			
Dementia subtype					
Alzheimer's disease	8 (30.8%)	4 (36.4%)			
Mixed dementia	2 (7.7%)	0			
Vascular dementia	4 (15.4%)	2 (18.2%)			
Lewy Body dementia	6 (23.1%)	4 (36.4%)			
Other	2 (7.7%)	1 (9.1%)			
Unknown	4 (15.4%)	0			
IMD Quintile^b^					
1 (least disadvantaged)	11 (42.3%)	6 (66.7%)	3 (23.1%)	2 (28.6%)	14 (43.8%)
2	4 (14.5%)	2 (22.2%)	3 (23.1%)	2 (28.6%)	2 (21.9%)
3	0	0	3 (23.1%)	2 (28.6%)	3 (9.4%)
4	3 (11.5%)	0	1 (7.7%)	0	4 (12.5%)
5 (most disadvantaged)	1 (3.8%)	1 (11.1%)	3 (23.1%)	1 (14.3%)	4 (12.5%)
Job role					
Activity Coordinator			1 (6.3%)	0	
Care home liaison			1 (6.3%)	1 (11.1%)	
Care quality			1 (6.3%)	0	
Care assistant			4 (25.0%)	3 (33.3%)	
Senior care assistant			2 (12.5)	0	
Night care assistant			1 (6.3%)	1 (11.1%)	
Housekeeper			1 (6.3%)	1 (11.1%)	
Matron			1 (6.3%)	0	
Manager			4 (25.0%)	3 (33.3%)	
	Mean (SD), [Range]				
Age^a^	62.3 (±9.5) [42–89]	61.1 (±5.2) [51–68]	41.8 (±16.6) [18–62]	43.3 (±17.2) [21–60]	54.8 (±15.9) [18–89]
Years of education	17.9 (±2.9) [11–23]	18.09 (±1.5) [16–20]	15.7 (±2.7) [11–20]	16.4 (±2.6) [11–19]	17.1 (±3.0) [11–23]
Care home capacity	41.5 (±17.4) [18–76]	38.9 (±18.2) [18–76]	42.2 (±15.8) [12–64]	49.7 (±11.6) [36–64]	41.7 (±16.6) [12–76]
Years working in a care home			9.3 (±10.6) [1–35]	7.0 (±11.1) [1–35]	
Years since dementia diagnosis	6.7 (±3.6) [2–16]	7.0 (±4.4) [2–16]			
Years (PLWD) residing in a care home	2.7 (±2.1) [1–10]	2.8 (±1.9) [1–7]			

^a^ *n* = 1 care home staff = prefer not to say.

^b^ *n* = 4 missing data.

**TABLE 2:** Coding tree of the themes and sub‐themes conceptualized following thematic analysis

Theme	Subtheme
1: Developing anger and frustration	Escalating negative emotions towards care homes and loss of trust Families' relentless distress and upset, with no sign of relief Anger towards Government and loss of trust
2: Impact on relationships	Abandonment and guilt Relationship breakdowns
3: Stress and burnout	Care home staff burnout Need for staff support
4: Behavioural changes, and perceived impact on residents	—

### 3.1 THEME 1: Developing anger and frustration

#### 3.1.1 Escalating negative emotions towards care homes and loss of trust

Over the course of the data collection, between baseline and follow‐up interviews, family carers became increasingly angry and frustrated at care homes, particularly in relation to visiting restrictions. Prior to the rollout of vaccines and increased testing (baseline interviews), family carers were frustrated and upset about not having seen their relatives in a long time, or only infrequently through window visits or via digital/virtual platforms (e.g. skype/facetime). However, the emotional intensity had amplified at the follow‐up interviews, as family carers became angry at the continued inability (or reduced ability) to visit their relatives face‐to‐face despite having been vaccinated and adhering to restrictions.

they [care staff] put a portacabin in the car park so…it's not attached to the home in any way…and it had 2 entrances, 1 for the residents and 1 for the visitor and…I can't get in my head why we can't be using that? It [visiting the care home] is crazy it really is. I've got to the point now where I'm thinking they [care staff] just don't want us to come back and visit, it's too much trouble. **F‐Up ID01, female carer, spouse**

This anger was further exacerbated by the fact that multiple care home staff could be in close proximity to the resident, with family carers not allowed. As most family carers were not allowed to enter the care home and have face‐to‐face visits, yet COVID‐19 was spreading in some care homes, family carers lost trust in the systems, fuelled by the knowledge that care home staff were most likely the axis on which COVID‐19 entered the care home.

when I Skype my mum … I've counted 20 different carers sitting with her and you think well why can't I do that if 20 carers can sit there but me, who doesn't go anywhere or doesn't do anything … I'm not allowed to go in to see my own mother and do the same thing, it just doesn't make any sense. **F‐Up ID20, female carer, daughter**

### 3.2 Families' relentless distress and upset, with no sign of relief

The continued inability to see their relatives and communicate effectively with them during the pandemic led some carers to seek medical/psychological support. With no support or changes in practice from care homes, the distress experienced by family carers increased over the course of the study.

and then the restrictions came in and I had that very long period of not seeing him at all. Now a couple of times literally twice I saw him through his bedroom window and he became very excited very agitated, trying to get at me, trying to run through the doors to try and get round to touch me and hug me and of course he couldn't. So that was very distressing, so that was about 22 weeks that we didn't see him for and during that time the home did try zoom and Facetime but he can't manage the technology. **ID11, female carer, spouse**

I'm now getting counselling because I can't cope with it. I don't have any other family here I'm totally on my own, I live on my own. F‐Up **ID01, female carer, spouse**

At the time of follow‐up interviews, family carers reported greater levels of seeking support and advice from various networks including peers, health professionals, or through medication use and counselling, to tackle their ongoing feelings of stress and grief.

I have 2 friends who are GPs, 1 of them's retired now we meet up every Saturday on a Zoom call and err we sort of have counselling session…my friend's father is in a similar position, he's in a care home and her mother hasn't been allowed to see him at all for 12 months **F‐Up ID20, female carer, daughter**

what I worry about with mum if we all go in and then they say oh no we are closing it down again it's like oh I've got to go through all this again…I do suffer, I suffer terribly I don't sleep well…I'm still on the good old anti‐depressants…and I have a counsellor ring me and I can ring her any time and she's really good but yeah, it's just one of those things isn't it it's just what's happening at the moment, it's just a horrible time **F‐Up ID10, female carer, daughter**

### 3.3 Anger towards government and loss of trust

Family carers were also angered and frustrated at the government and the lack of central guidance to enable safe care home visitation. Family carers missed out on important time with their relatives living with progressive and exacerbating dementia. The lack of clear guidance across all care homes led to variations in visiting rights between family carers, with some noting their friends and peers being able to visit relatives in care homes, yet themselves being unable to. This inequity further fuelled the family carers' emotional upset and caused increasing distress throughout the pandemic.

I'm a bit angry, I'm a bit angry with the government really and in another way, I can see that they've got to keep everybody safe, so I get that, but then from a personal point of view, I get angry that I've missed out on that time, you know my friends' parents, I've got three friends and their three parents they were able to sit with them when they were ill and nurse them and I just couldn't, I just couldn't get near and it was the worse feeling in the world. **F‐Up ID10, female carer, daughter**

Additionally, the reportedly inequitable vaccination rates between staff and family carers further exemplify a lack of clear central strategy and guidance. As a result of this continued lack of support and guidance, family carers quickly lost trust in the government and described new emotions of anger and frustration, which were not identified in the baseline interviews, but developed by the point of follow‐up.

### 3.4 THEME 2: Impact on relationships

#### 3.4.1 Abandonment and guilt

Many family carers expressed feelings of guilt for having moved their relative into a care home during lockdown, or for being unable to visit them in the care home since restrictions took a hold. While restrictions were imposed by the care homes, family carers attributed the blame on themselves for not providing the care and spending the time they normally would with their relative. Many carers felt as if they had abandoned their relative.

we've abandoned her and I was sort of feeling obligated really in a lot of ways to go every week at least just so my mum knew we were there so (21.13) the pandemic but I could no longer do that and it was quite stressful for me to feel sort of like I should be going to see her and I couldn't. **ID03, female carer, daughter**

It's been difficult for me because I'm losing contact with her and so I feel guilty I suppose in not being more hands on. **ID07, male carer, spouse**

know the whole thing is bizarre but its also exceptionally distressing and although this sounds a bit dramatic I have this overwhelming feeling that I've abandoned him. **ID11, female carer, spouse**

These feelings were heightened when carers had alternative face‐to‐face visits in the care home, such as a window or pod visit, and then witnessing the upset and lack of understanding in their relative as to why they were unable to see each other In close proximity without any glass screens. This caused upset in carers and exacerbated feelings of guilt. Some carers felt they should ideally be able to care for their relative at home, but the increased caring demands of advancing dementia and the lack of social care support in the community since the pandemic outbreak, was beyond the capabilities of many, who then had to fast‐track care home admission of their relative.

my wife got very upset about this she because she didn't understand. She was talking about COVID it's like talking to a child and she was very very upset and now that obviously upset me. Now my son was with us and he ended up having to walk away from her he walked away from the window, he couldn't stand watching her because she was it was upset. **ID05, male family carer, spouse**

The lack of understanding from people living with dementia was also noted by care home staff as adding to the relationship strains between family and residents. With many residents living with dementia unable to understand why family was no longer visiting, they experienced feelings of neglect, according to care home staff.

at the start of this pandemic there were some residents that have dementia and they didn't really understand what was going on, they thought their family members like just didn't like them anymore. **ID13, female care assistant**

### 3.5 Relationship breakdowns

While PPE is important to wear for all staff, face masks in particular were found to cause difficulties for some staff in maintaining their positive and caring relationships with residents. Face masks were hiding facial features, rendering some staff unrecognizable to residents living with dementia, as well as obscuring facial expressions and muffling the voice, and thus basic communication. According to some carers, this lack of visual facial cues had an impact on the well‐being of residents.

it just got to be like a happy lounge into this blanket of sadness and depression and when you're wearing facemasks as well residents can't see that smile and you're unrecognisable. **ID32, female activity coordinator**

Some staff also expressed their frustration at the inability and lack of visitation from family carers, and the fact that they were supposed to oversee all family‐resident interactions to ensure guidelines are adhered to. One member of staff in particular described this as akin to prison‐like behaviour, and felt uncomfortable watching over all interactions. This then impacted on staff’ well‐being in their job, and made the workplace difficult to work in.

and now and then we had to kind of keep an eye on them which was horrible ‘cause it kind of felt like you're a prison warden or something. **ID13, female care assistant**

Relationships between staff and families equally broke down during the pandemic, long‐term imposed restrictions, care home closures. Family carers were missing updates about their relatives from staff, often experienced difficulties trying to talk to staff over the phone, and were generally not engaging as much with staff as in pre‐pandemic times due to lack of face‐to‐face visits. In addition, the inability for most to have face‐to‐face visits yet seeing a number of different members of staff behind the window or pod screen sitting next to their relative further caused anger in family carers and further broke down existing relationships or hindered the build‐up of relationships with staff.

my argument all along through this has been that I don't see myself as the risk to my mum…the staff are the risk because they're coming and going on a daily basis…and I know they're getting tested weekly but it's the staff that are bringing COVID into the home because, because none of the families have been able to get in there so it feels, so it's a little bit frustrating to say the least…and I've had the first dose of my vaccine now, my mum's had her first dose…we've been told that the likelihood is things aren't going to change in the care home. **FU‐up ID29, female carer, daughter**

the only thing that ever gets put in writing comes from Head Office we never get anything from the care home itself. So the Head Office who have 120 care homes I believe send a generic letter to all relatives telling us the same thing erm and basically it says in order to protect our residents and colleagues this is what happens you know this is why we can't have visits. Erm its it is the colleagues who are the problem you know it's it is crazy it really is. I've got to the point now where I'm thinking they just don't want us to come back and visit it's too much trouble. **F‐Up ID01, female carer, spouse**

### 3.6 THEME 3: Stress and burnout

#### 3.6.1 Care home staff burnout

Staff reported increased workloads and burnout from the changing demands of caring for residents during the pandemic, managing additional tasks such as testing and procuring PPE, as well as managing alternative care home visits and enabling contact between residents and family carers. Given the nature of the pandemic, staff reported high levels of staff sickness on many occasions, which left staff on shift having to cover for missing staff and taking on additional care tasks. In addition to greater and varying demands on staff, time spent working appeared to have increased overall for many, fuelling the burnout experienced.

Coping with residents' deaths from the virus, as well as the anxiety about the virus in general, added to increased levels of stress and burnout in staff, providing a broad range of issues faced by this group.

You know it's increased stress levels and increased our work. Whereas previously if I was working say Monday to Friday I could switch off. And through an on‐call system we'd manage the weekend but now I'm pretty much working seven days a week. **ID18, male care home manager**

As part of the additional tasks, some care home staff took it upon themselves to update families about the well‐being of their relative. One member of staff specifically highlighted how she wanted to support families better by providing regular phone calls to them, and thus provide a link between families and residents. However, these additional tasks and staff demands can also add to the burden and stress experienced in care home staff.

then the families are saying they're getting no calls…I just took it all on myself because we were a family and I thought I'd want someone to do this for me if it was my mum or dad so…I was just trying to make things right for everybody. **ID32, female activity coordinator.**

#### 3.6.2 Need for staff support

A very limited number of staff reported having received access to support for their mental well‐being during the pandemic. Care home managers appeared to be aware of the strains that the pandemic and its additional job demands have placed on their staff, while also highlighting the emotional impact of living through the pandemic and having family at home and caring for them. In some cases, staff were also constrained in seeing their own families outside work. Managers themselves expressed a need to ensure that staff mental health is being looked after.

In terms of staff, you have to ensure their mental health wellbeing, and they are mentally and physically well throughout, because they've got restrictions, obviously not only in the service but outside in their homes as well. So their engagement with people that they love is limited as well, so that has a huge impact on their emotional wellbeing. You have to be positive because you know peoples' lives are in your hands and you've got moral of individuals that work for you, to keep their moral, because despite what's actually happening within the care home you have to think of them as well, and they've got their own issues with families where they're not being able to see people. **ID16, female care home manager**

I think there's been a step up in national access to training events, online events for staff and carers particularly recently. Especially in light of the trauma that staff have experienced through death and caring for people who are dying you know. That's stepped up and then the support to staff, whether it's through a dedicated line from Samaritans or elsewhere because we're just a small organisation. You know we're not North West wide or national or international organisation with huge HR teams that you know can provide a wellbeing service for staff. The fact that our local contracts team have pulled together a resource for our staff has been so welcome. Because it has been traumatising. **F‐Up ID18, male care home manager**

One way care home managers tried to actively protect the mental health and well‐being of staff was promoting the ‘Care Workforce’ app. This was not discussed by all managers or staff though, with many staff lacking any form of psychological support during the pandemic.

I made sure they've all got the care workforce app and its downloaded on to all of our work devices so they've got access to it like that and they can do meditation and yoga, setting up resilience and wellbeing classes and breathing exercises and meditation. So, we're trying to look after everybody in a whole. **ID41, male care quality manager**

### 3.7 THEME 4: Behavioural changes, and perceived impact on residents

Many residents were observed to be upset and emotionally impacted by the sudden and prolonged lack of visitation at the beginning of the pandemic, as well as the continued infrequent visits later on. Staff reported the emotional impact this had on residents and their well‐being, witnessing deterioration in mental well‐being on a daily basis. Family carers, however, were only witnessing this distress during alternative face‐to‐face visits or remote visits, or hearing about it when staff relayed this information to families, which was not often the case.

Staff themselves felt distressed on seeing residents with dementia cry and be upset about being unable to see their families. For residents with dementia it was then very difficult for staff to repeatedly explain why things had changed and why they had to stay inside the care home without (much) family contact. Going to work on a daily basis witnessing deteriorating levels of mental well‐being in residents also took its toll on staff.

why tell a family like your loved one is absolutely beside herself sobbing every day, that's not going, so they don't know so this poor lady or gentleman they ended up going on antidepressants because they're crying and getting all upset that's affecting the people who are sitting around them so they're just sitting there drugged up basically to keep them calm and you think that lady was before lockdown she was up dancing with me. **ID32, female activity coordinator**

biggest thing was the lack of visitors, visiting stopped immediately and I noticed that we call them the service users they tended to start getting really upset and more so confused than what they were that they felt like they were a bit abandoned and couldn't understand why even though you try to explain to them as best why you could. **ID42, female care assistant**

In contrast to most care home staff and family carers' reports on the well‐being of residents, some care home staff reported that residents seemed more settled since the lockdown, due to the lack of visitation and social stimulation from family and friends. This suggests however that residents were passive and not stimulated, and experienced a decline in cognition.

what I was saying is a lot of the managers that I've spoken to that worked in the dementia care homes were saying at the beginning of the lockdown and the first few weeks how much more settled the residents where and they were having less PRN medication being administered because there wasn't a lot of visitors going in and the homes were more quieter and more settled so I had a lot of comments from the managers to say that the residents were more settled. So I know it sounds a bit strange because obviously there's, I know with the media now there's a lot of relatives upset because they can't go and see their family if they're in a care home but not all, people that have got the capacity and are aware of what's going on would be miss their relatives, miss the visits and things like that but the people with dementia. From what the care homes were saying, the managers were saying they become more unsettled when they get visitors **ID34, female care liaison lead**

Some staff enabled a greater deal of alternative visits at the care home when they noticed the residents' mental health deteriorate substantially. Without guidelines provided for this, they made those decisions, enabling more families to see their relatives.

So I've noticed her deteriorating when I've been to see my mum I've noticed her in the in the lounge slumped over and just totally disengaging with people which isn't normal for her and I noticed her daughter going in about I don't know 2 months ago and she said that the manager had said you need to come in and start you know because mentally she's really deteriorating. I think through loss of contact and now she was she's been going in and obviously following a negative LFT and everything and now the difference in this lady is unbelievable, she's almost back to how she was before. **F‐Up ID21, female carer, daughter**

On some occasions, family carers were caught on the other side of this decision‐making process, with one family carer remarking that he saw one family go and visit their relative more frequently than he was allowed to visit his relative.

…at the moment we're still allowed, myself and my wife to go [into the home] separately…my brother who lived in [foreign country] has come back to live in this country again now so I've also contacted [the care home] and said well you need to let him as well now, which fair enough they have…although singularly. But then we've seen other people going in in twos, so they're bending the rules to suit, which I find quite frustrating to be honest **F‐Up ID29, male carer, son**

This again caused further aggravation from family carers towards care homes and their staff, given the perceived inequity of some family carers being allowed to visit more often than others.

## 4 DISCUSSION

This longitudinal qualitative study appears to be the first to have illustrated the emotional journeys that care home staff, residents and families have experienced throughout the COVID‐19 pandemic, in the UK specifically. All groups had been significantly affected, and relationships had been, and remain, severely strained by the pandemic, with ongoing needs for easily accessible mental health support highlighted for all groups interviewed.

In capturing the experiences over the first year of the pandemic, and its associated care home restrictions, the mental health and well‐being of all three groups—family carers, residents living with dementia and care home staff—appeared to have been affected detrimentally. Family carers in particular were shown to grow increasingly angry and emotionally upset about the lack of safe visitation to their relatives at the care homes. This corroborates quantitative survey findings from over 200 informal carers of care home residents in Ireland (O'Caoimh et al., 2020), with carers reporting lower levels of emotional well‐being as a result of the restrictions. Paananen et al. (2021) similarly reported the negative impact of the restrictions on family carers of care home residents, while equally highlighting the breakdown in relationships between families and staff. Family carers never stop caring, and often feel guilty for having placed their relative into a care home. Considering the large hours of unpaid care that many family carers of people living with dementia provide when their relative resides in the community (Wittenberg et al., 2019), suddenly leaving all care to a care home can be cause for distress in its own right. Reports of deterioration in the residents' mental well‐being, along with previous evidence of general deterioration in people's dementia symptoms both in the community and in care homes (Giebel et al., 2021; Tujit et al., 2021), may have exacerbated family carers feelings of guilt for their relative's mental and physical health decline.

With participants followed through a time of vaccination rollout and increased testing, family carers' emotional upset was fuelled by the inability to see their relatives face‐to‐face, despite being fully vaccinated. COVID‐19, however, was still entering some care homes and thus the virus is likely to have been brought in by staff, rendering it further incomprehensible to family carers that they should not be allowed in. Research into COVID‐19 transmission in staff supports this notion, with Ladhani et al. (2020) reporting a threefold higher risk of COVID‐19 positivity in care home staff working in multiple care homes, compared to staff only working in one care home. This becomes further problematic as many cases of COVID‐19 remain often undetected in care homes (Jeffrey‐Smith et al., 2021), supporting the need for care home staff to be fully vaccinated and for consistent testing of all involved.

A perception of poor mental well‐being within the care home residents was reported by the family and care workers, which coincides with previous evidence showing the detrimental impact of COVID‐19 restrictions on dementia symptoms (Giebel et al., 2021). However, families themselves further reported impacts to their own metal well‐being, and the care staff were equally affected, albeit for different reasons. Increased workloads and job demands, combined with a lack of guidance in how to provide care during the pandemic all contributed to staff feeling overburdened and burned out. Some managers who were interviewed reported providing support for their staff, while support did not seem to be available to all staff. This corroborates and further expands on in detail preliminary findings from nursing home staff in the United States. White et al. (2021) reported some of the emotional impacts on nursing home staff working during the pandemic, including fear of getting infected and infecting family members, high levels of burnout from increased job demands and emotional upset in dealing with residents who are socially isolated. Thus, the present findings further show how care home staff in different countries are emotionally affected by pandemic‐related changes to working demands, care practices and work environment. Even prior to the pandemic, care home staff were found to experience higher levels of burnout and stress when unsatisfied with their job, working in poor care home environments, feeling unsupported, lacking leadership, as well as working with residents with higher levels of behavioural problems (Costello et al., 2018; White et al., 2020). Considering the additional level of burden that the pandemic and its new ways of working have created, and the emotional upset of working in a sector which has been disproportionally affected by COVID‐19 deaths (ONS, 2021), there is an urgent need for adequate mental health support for care home staff, and likely social care staff more broadly.

Mental health support is thus required for all groups affiliated with care homes—staff, residents and families. Support particularly suitable for care professionals might be regular peer gatherings reflecting upon the job, such as via Schwartz Rounds (Farr & Baker, 2017), as well as psychological therapy or mindfulness techniques, with staff needing to be allocated the time to devote to this. Another way in which this might be achieved could be by rebuilding the relationships among the groups—the relations and trust that broke down between family carers and staff, as well as the relationships between staff and residents and family carers and residents. In the UK, face‐to‐face care home visits are allowed again, supported by a large vaccination rollout across Society. This means that relationships between family carers and residents may be part mended by regular visitation. However, family carers noted that the time they were left out of the care home they will never get back, as their relative had deteriorated further in their dementia, causing difficulties for re‐building these relationships. Engagement between family carers and staff may be rebuilt also by more regular contact, with family carers now allowed to go into the care home without restrictions in most cases, although further research needs to determine whether that is actually the case. Having more face‐to‐face contact with staff, as opposed to being reliant on occasional phone calls, newsletters, or no contact at all and only seeing staff behind the window or pod screen with their relatives, may bring some balance to the increasingly negative relationships that have built up during the pandemic, as also reported in other research (O'Caoimh et al., 2021; Paananen et al., 2021). It may further be beneficial for care homes to actively set up meetings or drop in sessions for family carers to talk to staff about the residents and to create a clear and positive, transparent line of communication between the two groups that are caring for the resident—family carers and staff.

### 4.1 Limitations

While this longitudinal qualitative study benefitted from a large (in qualitative research terms) sample from across the UK, including each staff recruited from a different care homes, there were some limitations. We had very limited minority ethnic representation, and only among staff. Research has highlighted how people from minority ethnic backgrounds have been at greater risk of COVID‐19 infection and mortality (Rose et al., 2020), and it would thus be important for future research to particularly explore their emotional experiences of working in, living in and caring for someone living in a care home. However, many families from minority ethnic backgrounds tend to care for their relatives at home as opposed to utilizing care homes (Nielsen et al., 2021) so that recruiting family carers from minority ethnic backgrounds for this purpose is more difficult.

## 5 CONCLUSIONS

The pandemic has had a detrimental impact on the lives of those surrounding care homes—from residents and staff to family carers. While care homes have reopened, vaccine rollout and increased testing improved and slowly enabled safe care home visiting, the impact on people's mental health and well‐being, and particularly strained relationships between family carers and care home staff, will take a long while to be mended and require urgent attention. Mental health support appears to be limited amongst staff, with none reported specifically for residents and family carers. This lack of support adversely impacts well‐being and the subsequent care of residents. Consideration should be given about how to best meet the needs for mental health support to all three groups, and approaches explicitly outlined in the new plans for social care recently announced by the UK government; for example, enhanced links between primary care provision and care homes as a mechanism for facilitating signposting and referral for residents and relatives to occupational health or NHS support for staff. Organizations should be encouraged to facilitate access to multiple opportunities of emotional support for staff and relatives in times of crisis, to manage the workload stresses associated with the role and thus maintain the best quality of care for residents and their families.

## CONFLICT OF INTEREST

None.

### PEER REVIEW

The peer review history for this article is available at https://publons.com/publon/10.1111/jan.15181.

## Supporting information


APPENDIX S1

APPENDIX S2

## Data Availability

The data that support the findings of this study are available on request from the corresponding author. The data are not publicly available due to privacy or ethical restrictions.
