# Supplementary material for: Guilt, tears and burnout—Impact of UK care home restrictions on the mental well‐being of staff, families and residents
Source: J Adv Nurs. 2022 Feb 21;78(7):2191–202. doi: 10.1111/jan.15181 (PMC9303866; doi:10.1111/jan.15181)
Supplement: Supplementary file 1 — APPENDIX S1 [file JAN-78-2191-s002.docx]

**APPENDIX I. Baseline Topic Guides**

**- Family carers -**

Question 1. Please tell us about your caring background, who you are caring for, and since when your relative has been residing in a care home?

Question 2. Has your relative entered a care home before or since the COVID-19 pandemic? What were the reasons for her/him entering a care home, and this particular care home? And what have your experiences been with regards to the care they have received?

*PROMPT: Was the initial 2-week quarantine considered at the time of care home entry during the pandemic?*

Question 3. If relevant, before the pandemic, how often were you able to visit your relative in the care home? How has that changed since that pandemic started?

Question 4. How did your relative react to your visits/to the lack of visitation (where applicable), and what do you think has happened as a result of the changes of visiting arrangement changes since the pandemic for them?

*PROMPT: Have the restrictions affected your relationship with your relative?*

Question 5. What have been the effects of the public health restrictions on your life as a carer more widely and what are your feelings about these restrictions?

Question 6. How has your relative’s life changed in the care home since the pandemic? Are they aware of the changes and are they socially engaging?

Question 7. How do you feel about the long-term implications of the pandemic on the ability to visit your relative and their well-being?

Question 8. How has the pandemic altered how your relative accesses medical care and hospital appointments whilst being in the care home?

Question 9. How has the care home communicated updates in public health information regarding access to your relative? Have they communicated via email or text message or do you have to contact the home for the latest arrangements?

Question 10. And finally any other key points we’ve missed that you’d like to tell us about?

**- Care home staff -**

Question 1. Please tell us about your regular working day before the pandemic?

Question 2. How has the pandemic affected your working practices? What do you consider should be being used and supplied in terms of PPE and do you have adequate PPE? Are supplies reliable?

Question 3. What measures has your care home taken since March to reduce risks to staff and residents during the pandemic? Have restrictions changed over time? If so why and how?

Question 4. How do you feel about working in a care home? In what ways if any has the pandemic changed your views?

Question 5. How has the general day-to-day life changed for care home residents? How do residents engage socially with fellow residents, in social activities, and family carers?

Question 6. What have your experiences been of facilitating interaction between residents and family carers?

Question 7. What have been the protocols around testing staff and residents? Have the results of tests been communicated quickly? What has happened as a consequence of a staff member or resident testing positive for Covid?

Question 8. What arrangements have there been for residents on respite? Have new residents come for long-term placements?

*PROMPT: How did they cope with the initial quarantine?*

Question 9. What do you think the future holds for your residents, their loved ones, and staff? What about your future in this field of work, and the care sector in general looking forwards?

Question 10. And finally any other key points we’ve missed that you’d like to tell us about?
