# Supplementary material for: Guilt, tears and burnout—Impact of UK care home restrictions on the mental well‐being of staff, families and residents
Source: J Adv Nurs. 2022 Feb 21;78(7):2191–202. doi: 10.1111/jan.15181 (PMC9303866; doi:10.1111/jan.15181)
Supplement: Supplementary file 2 — APPENDIX S2 [file JAN-78-2191-s001.docx]

**APPENDIX II. Follow-up interviews**

**- Family carers -**

**Question 1.** Since we last spoke to you in autumn last year, what, if any, changes have there been to your caring situation or your relative living with dementia?

**Question 2.** Since we last spoke to you what, if any, changes have there been in visitation to the care home where your relative resides?

**Question 3.** Have you or your relative been vaccinated yet? If yes, what, if any, difference has this made to visiting permissions and arrangements to see your relative?

**Question 4.** If you have been able to visit your relative at the care home since autumn, how were these visits undertaken? How did you feel about the visits – were they positive or negative?

**Question 5.** What about the communication, advice and support you have been getting from the care home more generally? And what about government communication and guidance?

**Question 6.** What about the future- how do you predict things may change looking forward into 2021 and beyond?

**Question 7.** Can you tell us more about how the care home has updated relatives on the vaccination?

**Question 8.** Moving forward (post vaccine), how do you envisage the visitations rights to change, bearing in mind vaccination does not give full protection?

**Question 9.** Finally, is there anything else you feel you wish to share that we haven’t covered yet?

**- Care home staff -**

**Question 1.** Have you been vaccinated? If so, when was this? If not, why not?

**Question 2.** How has the uptake of vaccination been at your care home for both staff and residents? Who delivered the vaccines? What were your experiences surrounding the vaccination process?

**Question 3.** Since last autumn, has your role changed or are you still in the same role?

**Question 4.** Since last autumn, have there been any major changes in how you are delivering care at the care home?

**Question 5.** Have you faced any further difficulties in delivering care during the pandemic? PROMPT: Access to PPE, testing, vaccination

**Question 6.** How have family visits been taking place since we last spoke? Have testing and/or vaccination supported safe visitation?

**Question 7.** Has communication from the government surrounding care home visitation advice changed and has it been consistent?

**Question 8.** How do you now feel about the future of care homes and especially visitation of family members?

**Question 9.** And finally, any other key points we’ve missed that you’d like to tell us about?
